# Genomic characterization of JG068, a novel virulent podovirus active against *Burkholderia cenocepacia*

**DOI:** 10.1186/1471-2164-14-574

**Published:** 2013-08-27

**Authors:** Karlene H Lynch, Ashraf H Abdu, Max Schobert, Jonathan J Dennis

**Affiliations:** 1 Department of Biological Sciences, 6–008 Centennial Centre for Interdisciplinary Science, University of Alberta, Edmonton, AB T6G 2E9 Canada; 2 Institute of Microbiology, Technische Universität Braunschweig, Spielmannstr. 7, 38106 Braunschweig, Germany

**Keywords:** *Burkholderia cepacia* complex, Phage therapy, *Autographivirinae*, ϕKMV-like phages, SAR endolysin, *Galleria mellonella*

## Abstract

**Background:**

As is true for many other antibiotic-resistant Gram-negative pathogens, members of the *Burkholderia cepacia* complex (BCC) are currently being assessed for their susceptibility to phage therapy as an antimicrobial treatment. The objective of this study was to perform genomic and limited functional characterization of the novel BCC phage JG068 (vB_BceP_JG068).

**Results:**

JG068 is a podovirus that forms large, clear plaques on *Burkholderia cenocepacia* K56-2. Host range analysis indicates that this phage can infect environmental, clinical, and epidemic isolates of *Burkholderia multivorans*, *B. cenocepacia*, *Burkholderia stabilis*, and *Burkholderia dolosa*, likely through interaction with the host lipopolysaccharide as a receptor. The JG068 chromosome is 41,604 base pairs (bp) in length and is flanked by 216 bp short direct terminal repeats. Gene expression originates from both host and phage promoters and is in the forward direction for all 49 open reading frames. The genome sequence shows similarity to *Ralstonia* phage ϕRSB1, *Caulobacter* phage Cd1, and uncharacterized genetic loci of blood disease bacterium R229 and *Burkholderia pseudomallei* 1710b. CoreGenesUniqueGenes analysis indicates that JG068 belongs to the *Autographivirinae* subfamily and ϕKMV-like phages genus. Modules within the genome encode proteins involved in DNA-binding, morphogenesis, and lysis, but none associated with pathogenicity or lysogeny. Similar to the signal-arrest-release (SAR) endolysin of ϕKMV, inducible expression of the JG068 SAR endolysin causes lysis of *Escherichia coli* that is dependent on the presence of an N-terminal signal sequence. In an *in vivo* assay using the *Galleria mellonella* infection model, treatment of *B. cenocepacia* K56-2-infected larvae with JG068 results in a significant increase in larval survival.

**Conclusions:**

As JG068 has a broad host range, does not encode virulence factors, is obligately lytic, and has activity against an epidemic *B. cenocepacia* strain *in vivo*, this phage is a highly promising candidate for BCC phage therapy development.

## Background

In recent years, the emergence of antibiotic-resistant Gram-negative pathogens – including *Acinetobacter*, *Klebsiella*, and *Pseudomonas* – has become a serious global concern [1]. As the treatment options for these bacteria become increasingly limited, scientists and clinicians alike have turned to bacteriophage (or phage) therapy as a possible alternative to antibiotic delivery. By preventing and/or treating infections with phages – viruses that specifically infect bacteria – one can target pathogens that are resistant to conventional drug treatment while avoiding possible antibiotic side effects, such as disruption of the patient’s normal flora [2]. Phages infecting the Gram-negative bacteria *Escherichia coli* and *Pseudomonas aeruginosa* have already been shown to be safe for human administration in multiple volunteer and phase I trials [3-6]. Although relatively little data are currently available for controlled human efficacy studies, a recent phase I/II trial showed that *P. aeruginosa*-specific phages were clinically active [6].

The *Burkholderia cepacia* complex (BCC) is a group of antibiotic-resistant Gram-negative species that also appear to be a promising target for phage therapy. These bacteria cause transmissible and potentially fatal opportunistic infections in cystic fibrosis (CF) and immunocompromised patients. Similar to *P. aeruginosa*, BCC bacteria are innately antibiotic-resistant owing to a variety of mechanisms (reviewed in [7]). Antibiotics such as meropenem, minocycline, and ceftazidime show partial efficacy against some clinical isolates, but the vast majority of strains are not susceptible to even the administration of multiple drugs [8]. Although phage therapy for *Burkholderia* species has not yet reached clinical trials, preliminary studies have shown it to be safe and effective in the protection of crop seedlings and in both invertebrate and mammalian infection models [9-12].

One of the most important aspects of phage therapy development is the selection of appropriate therapeutic phage candidates. To be clinically applicable, a phage should have a broad host range (that includes clinical isolates) and a sequenced and characterized genome that lacks genes encoding putative pathogenicity factors and lysogeny-related proteins (reviewed in [13]). Identifying BCC-specific phages that meet all of the above criteria has thus far proven challenging, particularly with respect to lysogeny genes. Although several phages have been isolated and characterized that have a broad host range (encompassing multiple strains and species within the BCC) and no virulence genes, almost all of these phages are temperate or encode proteins required for this lifestyle [13,14]. BCC-specific phages that are obligately lytic (or putatively so) are thus far suboptimal for phage therapy development because they either infect only environmental isolates (Bcep1, Bcep43, Bcep781, and BcepB1A) [15] or have genomes that are either not sequenced or not published (KS12, BcepF1, BcepNazgul, BcepGomr, BcepEtu, BcepFife, and BcepBrny) [13,16]. Although we have shown that BCC phages can be engineered to an obligately lytic form, such mutants can be difficult to construct and could potentially encounter additional hurdles with respect to regulatory approval [11,17]. As a result of these obstacles, a key objective of BCC phage isolation and characterization studies is to isolate naturally occurring obligately lytic phages that infect clinical strains and lack putative virulence or lysogeny genes. Here, we describe and characterize the complete genome sequence of podovirus JG068, a novel phage possessing each of these characteristics. Furthermore, using a well-characterized invertebrate infection model [10,18], we show that this phage is active against *Burkholderia cenocepacia in vivo*, providing further evidence that JG068 and other related phages are appropriate candidates for clinical development.

## Results and discussion

### Isolation, host range, and morphology

JG068 (vB_BceP_JG068) was isolated from a sewage processing plant using an uncharacterized strain of *Burkholderia dolosa*. Additional JG068 hosts were identified using spot tests and soft-agar overlays of BCC strains. A total of 32 strains were tested and seven were found to support lytic propagation of JG068 (with efficiency of plating [EOP] values in parentheses): *Burkholderia multivorans* ATCC 17616 (10^-4^), *Burkholderia cenocepacia* K56-2 (10^0^), J2315 (10^-2^), and PC184 (10^0^), *Burkholderia stabilis* LMG 14294 (10^-1^), and *Burkholderia dolosa* AU0158 (10^0^) and CEP021 (10^0^). Excluding the soil isolate ATCC 17616, each of these strains was identified as a CF clinical isolate [19-21]. Furthermore, the three susceptible *B. cenocepacia* strains and AU0158 have all been linked to epidemic spread among CF patients [19,21]. This host range is relatively broad, clinically relevant, and distinct compared to the tropism of BCC phages that we have previously characterized [10,11,22-26]. On K56-2, JG068 forms large clear plaques, 1–3 mm in diameter.

Several BCC phages – including KS4, KS5, KS9, KS10, and KS12 – have been previously shown to use lipopolysaccharide (LPS) as a receptor [11], Abdu and Juárez Lara, unpublished data]. To assess if JG068 uses a similar receptor, a panel of both K56-2 [27,28] and PC184 [Abdu, unpublished data] LPS mutants were tested with the phage in spot tests. In K56-2 mutants, JG068 (propagated on wildtype K56-2 [EOP: 10^0^]) less efficiently infected *wbxE* and *waaL* mutants (EOP: 10^-4^) and did not infect *wabR*, *wabS*, *wabO*, or *waaC* mutants (EOP: <10^-6^; Table 1). In PC184 (EOP: 10^0^), this JG068 stock less efficiently infected a *wabP* mutant (EOP: 10^-2^) and did not infect *wabO* or *waaC* mutants (EOP: <10^-6^; Table 1). As JG068 does not infect mutants with significant deficits in the core LPS structure, the tail of this phage likely interacts with the LPS core of K56-2, PC184, and potentially other hosts. As noted in a previous BCC phage study, further experiments are required to validate this prediction as LPS truncation may also result in secondary changes to the cell surface structure of the mutants [11,27].

**Table 1:** JG068 LPS mutant host range

***B. cenocepacia *strain**	**Mutant strain**	**Mutation**	**Reference**	**Phenotype**	**EOP**
K56-2	wildtype	none	[19]	wildtype	10^0^
	RSF19	*wbxE*	[27]	truncated O-antigen	10^-4^
	XOA7	*waaL*	[28]	no O-antigen	10^-4^
	XOA15	*wabR*	[28]	truncated outer core	<10^-6^
	XOA17	*wabS*	[28]	truncated outer core	<10^-6^
	XOA8	*wabO*	[28]	truncated inner core	<10^-6^
	CCB1	*waaC*	[28]	truncated inner core	<10^-6^
PC184	wildtype	none	[19]	wildtype	10^0^
	Δ*wabP*	*wabP*	[Abdu, unpublished data]	truncated inner core	10^-2^
	Δ*wabO*	*wabO*	[Abdu, unpublished data]	truncated inner core	<10^-6^
	Δ*waaC*	*waaC*	[Abdu, unpublished data]	truncated inner core	<10^-6^

**Figure 1:**
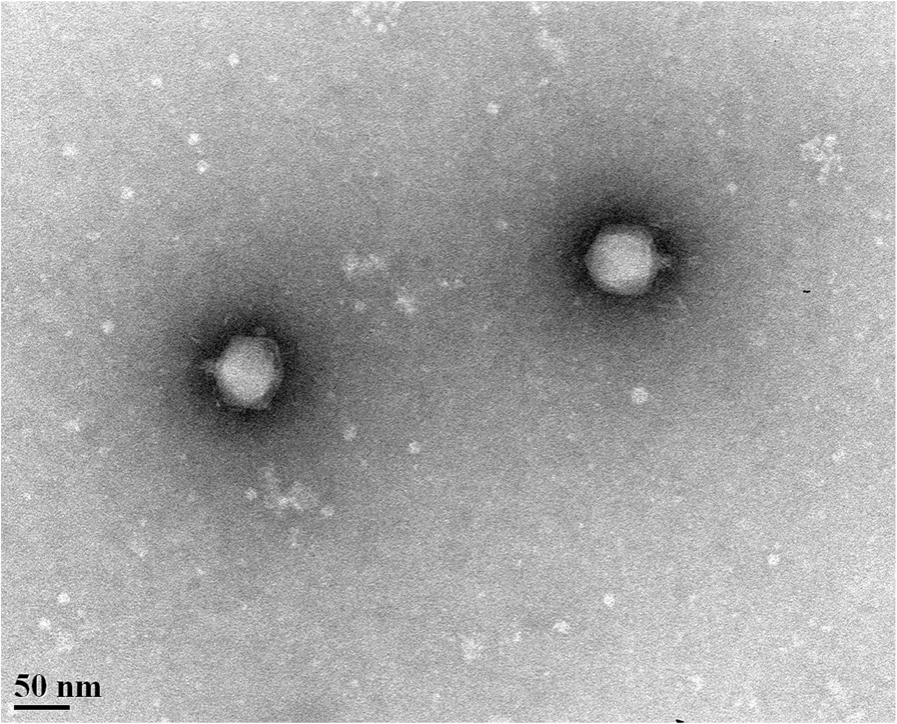
**Transmission electron micrograph of phosphotungstic acid-stained JG068 virions at 140,000-fold magnification.** Scale bar represents 50 nm.

Electron microscopy of JG068 virions (Figure 1) shows that this phage has a short tail and belongs to the C1 morphotype of the order *Caudovirales* and family *Podoviridae*[29]. This morphology is relatively rare for a BCC phage as the only *Burkholderia* podoviruses identified to date are the BPP-1-like BcepC6B and the Bcep22-like DC1, Bcep22, BcepIL02, and BcepMigl [14]. The JG068 capsid is icosahedral and 60.93 ± 2.83 nm in diameter (Figure 1).

### Genome sequencing and assembly

In order to determine if the genome sequence of JG068 was novel, several EcoRI genomic DNA fragments were cloned into pUC19 and sequenced. Resulting reads were analyzed using BLASTN and found to be similar (but not identical) to a variety of sequences, including those from previously characterized podoviruses. The genome sequence was completed using ion semiconductor technology on the Ion Torrent platform. A total of 1.07 × 10^5^ sequence reads were aligned into a single contig with over 400-fold coverage. Regions of ambiguity and genome ends were analyzed using Sanger sequencing.

The JG068 genome is 41,604 bp in length and has a 60.7% GC content. Based on the similarity of the sequence to characterized ϕKMV-like *Autographivirinae* genomes (discussed below), the chromosomal termini of JG068 are likely to be short direct terminal repeats (DTRs) as canonically found in T7 [30]. To identify these termini, JG068 DNA was directly sequenced using primers that bind the following four loci: within the left DTR, extending to the left terminus; immediately outside of the left DTR, extending to the left terminus; within the right DTR, extending to the right terminus; and immediately outside of the right DTR, extending to the right terminus. Primers that bind *within* the left DTR will amplify both the left and right ends of the genome simultaneously (as the primer binding site is in both DTRs). A reduction to one-half intensity in the sequence chromatogram represents the arrest of half of the reads (those originating from the left end of the genome) at the left chromosome terminus [31]. Primers that bind *immediately outside of* the left DTR will amplify only the left end of the genome (as the primer binding site is unique) and the sequence chromatogram will stop abruptly at the left chromosome terminus [30]. The same reasoning is true for primers binding within or near the right DTR. Using these methods, it was determined that the chromosomal termini of JG068 are 216 bp DTRs with an identical sequence on each end. The length of these repeats is shorter than that of other ϕKMV-like *Autographivirinae* such as LKD16 (428 bp), ϕKMV (414 bp), LKA1 (298 bp), and LIMElight (277 bp) [32-34].

In ϕKMV, genome replication is bidirectional with the origin and terminus in the DNA polymerase gene and an internal virion protein gene, respectively [33]. Using GenSkew analysis, a global co-minimum GC skew was identified in the JG068 DNA polymerase gene *18* and a global maximum skew was identified in the internal virion protein gene *38*, suggesting that the replication process of JG068 is similar to that of other ϕKMV-like *Autographivirinae*. In addition to the chromosomal termini and the putative replication origin, a third commonality with respect to the DNA of this genus of phages is that the JG068 genome lacks recognition sites for many common restriction enzymes, including BamHI, BglII, PstI, SacI, SmaI, and XhoI. As in other phages, including those that are ϕKMV-like, these sites tend to be lost as the phage evolves to avoid host restriction systems [32,33].

### Relatedness

When the sequence of JG068 is analyzed using BLASTN, the most similar sequences (with E-values of 2e^-87^ or less) are those of *Ralstonia* phage ϕRSB1, a genetic locus of blood disease bacterium R229, a genetic locus of *Burkholderia pseudomallei* 1710b chromosome 1, and *Caulobacter* phage Cd1. In the BLASTN results, JG068 shows similarity to several members of the *Autographivirinae* subfamily and ϕKMV-like phages genus, including ϕRSB1, Cd1, and *Pseudomonas* phage Bf7. Members of this subfamily (with enterobacteria phage T7 being the best characterized) belong to one of several genera, including the T7-like phages, SP6-like phages, ϕKMV-like phages, and novel genera that have not yet been named [35,36]. These podoviruses all encode a single subunit RNA polymerase, as is found in JG068 [35]. To determine if JG068 belongs to this subfamily (and, if so, to what genus), we used CoreGenesUniqueGenes (CGUG) analysis to compare the genomes of JG068, T7 (NC_001604.1), SP6 (NC_004831.2), and ϕKMV (NC_005045.1) [37]. Using T7, SP6, or ϕKMV as the reference genome, the proteins of JG068 were 15.0%, 23.1%, or 44.9% similar, respectively. As ≥40% similarity indicates a genus-level relationship [35], we can conclude that JG068 is a novel member of the *Autographivirinae* subfamily and the ϕKMV-like phages genus. As noted above, the few BCC podoviruses that have been previously characterized are either Bcep22-like or BPP-1-like [14], thus JG068 is the first BCC *Autographivirinae* phage to be identified.

### Genome annotation

**Figure 2:**
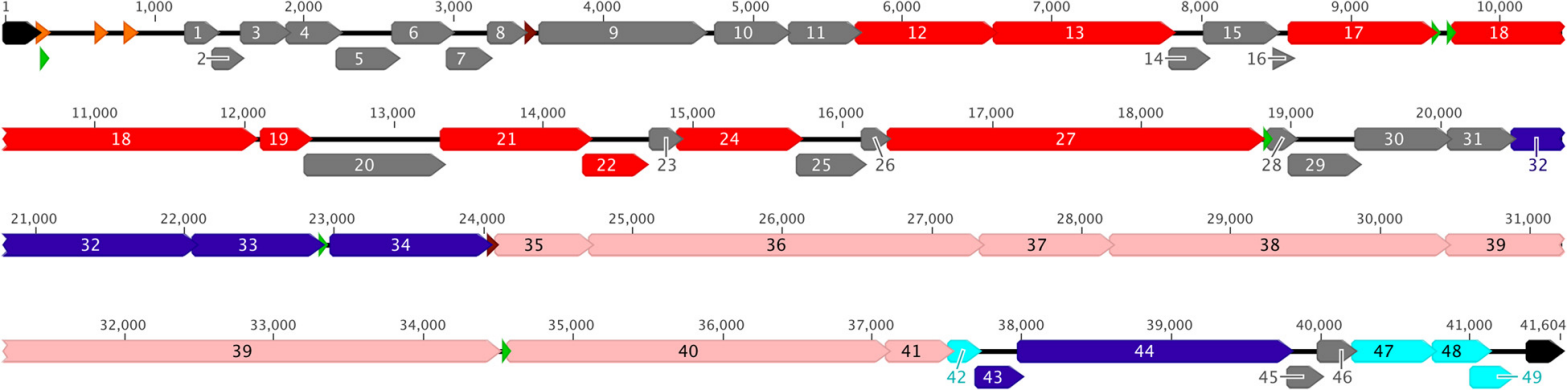
**JG068 genome map.** All genes (right-facing arrows labeled with the gene name [1-49]) are transcribed in the forward direction. Regulatory and repeat sequences: orange triangle, host promoter; green triangle, phage promoter; brown triangle, terminator; black, direct terminal repeat. Gene product functions: red, DNA-binding; purple, capsid morphogenesis and DNA packaging; pink, tail morphogenesis; blue, lysis; grey, unknown function.

The JG068 genome contains 49 putative open reading frames (ORFs) (Figure 2, Table 2). Similar to T7 and other *Autographivirinae*, all of the genes are transcribed in the forward direction [33,38]. Based on BLASTP analysis (with an E-value cutoff of 0.01), 19 JG068 proteins show no similarity to other proteins in the database (Table 2). The remaining 30 proteins have percent identities between 29% (Rz protein gp48, similar to hypothetical protein Bpse9_41836 of *B. pseudomallei* 91) and 64% (hypothetical protein gp8, similar to a hypothetical protein of *Ralstonia* phage RSB2) (Table 2), thus showing low-to-moderate similarity to other sequences at the protein level.

**Table 2:** JG068 genome annotation

**Gene**	**Start**	**End**	**Putative function**	**Strand**	**Putative ribosome binding site and start codon**	**Length (amino acids)**	**Closest relative**	**BLASTP alignment region (amino acids)**	**Percent identity**	**E-value**	**Organism**	**GenBank accession number**
*1*	1211	1405	hypothetical	+	ACGGAGctagacgaATG	64	none					
*2*	1402	1566	hypothetical	+	GGAACGAtgctcgATG	54	none					
*3*	1590	1889	hypothetical	+	GGAGTAAcgatcATG	99	hypothetical protein ϕAB1_gp1	3-72/169	50	1e^-13^	*Acinetobacter* phage ϕAB1	ADQ12705.1
*4*	1895	2227	hypothetical	+	GGGGTGAGttcgATG	110	none					
*5*	2224	2613	hypothetical	+	GGAGAAtcacGTG	129	hypothetical protein phAPEC8_0044	1-98/106	32	6e^-8^	*Escherichia* phage phAPEC8	AFU62620.1
*6*	2603	2974	hypothetical	+	GAGGTGttgaATG	123	none					
*7*	2971	3231	hypothetical	+	AACGAGGccgcATG	86	none					
*8*	3242	3460	hypothetical	+	AAGGAGcaacacATG	72	hypothetical protein	27-54/109	64	4e^-6^	*Ralstonia* phage RSB2	BAJ51800.1
*9*	3584	4672	hypothetical	+	AGGACAtcaATG	362	none					
*10*	4762	5223	hypothetical	+	AAGGAtttaacATG	153	none					
*11*	5252	5698	hypothetical	+	AAGGAActgacATG	148	none					
*12*	5702	6610	DNA primase	+	GGAGGctaaggcATG	302	putative DnaG-like primase	1-277/284	43	1e^-63^	*Caulobacter* phage Cd1	ADD21651.1
*13*	6624	7802	DNA helicase	+	GAAGGTAAcacgcaaGTG	392	hypothetical protein BURPS1710b_1647	35-427/431	61	2e^-176^	*Burkholderia pseudomallei* 1710b	YP_333051.1
*14*	7802	8032	hypothetical	+	GGGGGtgtgATG	76	none					
*15*	8032	8493	hypothetical	+	AAGGAGGGtgcgtgATG	153	none					
*16*	8493	8603	hypothetical	+	GTGGGGcgctgATG	36	none					
*17*	8600	9553	DNA ligase	+	GGAGGAAggtATG	317	PBCV-1 DNA ligase	4-309/309	37	1e^-41^	*Burkholderia pseudomallei* 91	ZP_02453445.1
*18*	9688	12060	DNA polymerase	+	AAGGAAccATG	790	38L	1-806/808	60	0	*Burkholderia pseudomallei* 91	ZP_02453448.1
*19*	12122	12424	HNH endonuclease	+	AGGCAcggtcgcagcATG	100	endonuclease	30-112/249	47	7e^-18^	*Bacteroides finegoldii* DSM 17565	ZP_05414969.1
*20*	12421	13314	hypothetical	+	AAGGAAcaagtATG	297	37L	8-260/276	50	7e^-71^	*Burkholderia pseudomallei* 91	ZP_02453449.1
*21*	13327	14301	DNA exonuclease	+	AAGGAGGcaaggccATG	324	phosphodiesterase I	1-247/258	48	5e^-75^	*Burkholderia pseudomallei* 1710a	ZP_04951398.1
*22*	14282	14677	DNA endonuclease	+	ATGAGAttgaccacaagcATG	131	DNA endonuclease VII	1-115/118	47	2e^-28^	blood disease bacterium R229	CCA83273.1
*23*	14727	14909	hypothetical	+	GGCAGGttgattgcATG	60	none					
*24*	14910	15704	DNA exonuclease	+	GGAGAttaaATG	264	34L	1-263/263	60	1e^-115^	*Burkholderia pseudomallei* 91	ZP_02453452.1
*25*	15704	16138	hypothetical	+	GGTGAGctaATG	144	hypothetical protein	16-128/138	33	5e^-6^	*Pantoea* phage LIMElight	YP_007002888.1
*26*	16142	16297	hypothetical	+	GACGGAGtaacagATG	51	none					
*27*	16313	18799	RNA polymerase	+	GAGGAcactgATG	828	DNA-directed RNA polymerase	6-817/818	51	0	*Burkholderia thailandensis* MSMB43	ZP_02468154.1
*28*	18861	19025	hypothetical	+	AAGGAAccgGTG	54	none					
*29*	19003	19443	hypothetical	+	AAGGAActgcgcgATG	146	hypothetical protein BDB_mp60445	1-143/145	35	5e^-18^	blood disease bacterium R229	CCA83279.1
*30*	19444	20058	hypothetical	+	AAGGAGAtttgaATG	204	none					
*31*	20068	20478	hypothetical	+	AAGGAGGGagcATG	136	none					
*32*	20488	22065	head-tail connector protein	+	AAGGAGAAgacATG	525	head portal-like protein from phage	9-494/512	50	1e^-165^	blood disease bacterium R229	CCA83282.1
*33*	22066	22908	scaffolding protein	+	AAGGAGcgtaaATG	280	scaffolding-like protein from phage	59-249/255	36	2e^-18^	blood disease bacterium R229	CCA83283.1
*34*	22991	24031	capsid protein	+	AGGAGGAAcctcaATG	346	major capsid-like protein	8-336/336	57	6e^-133^	*Ralstonia* phage RSB1	YP_002213721.1
*35*	24099	24716	tail tubular protein A	+	AAGGAGActgctATG	205	tail tuber protein A from phage	1-200/203	40	9e^-42^	blood disease bacterium R229	CCA83285.1
*36*	24726	27326	tail tubular protein B	+	AAGGAGGcattATG	866	tail tuber protein B from phage	2-858/858	48	0	blood disease bacterium R229	CCA83286.1
*37*	27339	28196	internal virion protein	+	AAGGGGGGtcagtcATG	285	hypothetical protein Bpse38_32600	5-199/301	37	8e^-22^	*Burkholderia thailandensis* MSMB43	ZP_02468144.1
*38*	28206	30446	internal virion protein	+	AAGGAGGtagcATG	746	hypothetical protein RSB1_gp36	1-361/785	30	2e^-42^	*Ralstonia* phage RSB1	YP_002213725.1
*39*	30458	34495	internal virion protein	+	AAAGGAGAAGtaaATG	1345	internal virion protein	9-1318/1333	30	2e^-131^	*Caulobacter* phage Cd1	ADD21673.1
*40*	34572	37097	tail fiber protein	+	AGGAGGcaacATG	841	BcepGomrgp19	307-539, 321-459/669	44, 31	3e^-44^, 2e^-5^	*Burkholderia* phage BcepGomr	YP_001210239.1
*41*	37107	37520	tail fiber assembly protein	+	AAGGAGGttttATG	137	hypothetical protein Bcep1808_1285	7-141/146	41	2e^-28^	*Burkholderia vietnamiensis* G4	YP_001119130.1
*42*	37524	37706	holin	+	AGGAGtaagtaATG	60	none					
*43*	37703	37987	DNA maturase A	+	AGGAGcaagctATG	94	conserved hypothetical protein from phage	6-79/96	44	2e^-11^	blood disease bacterium R229	CCA83292.1
*44*	37989	39788	DNA maturase B	+	GAGGCAttgatATG	599	TerL large terminase subunit-like protein	4-604/605	59	0	*Ralstonia* phage RSB1	YP_002213730.1
*45*	39788	39994	hypothetical	+	GGAGGAAgtaATG	68	none					
*46*	39998	40222	hypothetical	+	AAAGGAGtaattcATG	74	none					
*47*	40232	40753	SAR endolysin	+	AAGGAGGcagcATG	173	phage-type lysozyme	68-211/223	36	8e^-27^	*Xanthomonas* phage Xp10	NP_858975.1
*48*	40763	41119	Rz	+	AGGAGAtaaccATG	118	hypothetical protein Bpse9_41836	5-105/106	29	6e^-4^	*Burkholderia pseudomallei* 91	ZP_02453410.1
*49*	41016	41267	Rz1	+	AAGGGGAAGctgaATG	83	exported hypothetical protein	18-79/83	52	4e^-11^	blood disease bacterium R229	CCA83297.1

BLASTP hits with an E-value <0.01 were included in the table.

Similar to other phages, the JG068 genome has a modular organization. In both T7 and ϕKMV, class I early genes are clustered on the left end of the genome, class II genes for DNA-binding proteins are found centrally, and class III genes for virion morphogenesis and lysis are positioned on the right end [32,38]. The overall organization of JG068 is syntenic with both of these phages: left end genes encode hypothetical proteins, central genes encode DNA-binding proteins, and right end genes encode capsid morphogenesis and DNA packaging, tail morphogenesis, and lysis proteins (Figure 2). Using BTXpred analysis (to identify toxins) combined with BLASTP and HHpred comparisons (to identify other pathogenicity-associated proteins; Tables 2 and 3), no putative virulence modules were detected, indicating that JG068 is likely to be a safe candidate for clinical testing.

**Table 3:** JG068 HHpred predictions

**Protein**	**Motif of closest relative**	**Motif definition**	**Percent probability**	**E-value**
gp12	2au3_A	DNA primase	100	1.10e^-42^
gp13	3bgw_A	DNAB-like replicative helicase	100	1.70e^-48^
gp15	1v58_A	Thiol:disulfide interchange protein DSBG	93.88	0.13
gp17	1fvi_A	Chlorella virus DNA ligase-adenylate	100	3.90e^-60^
gp18	3pv8_A	DNA polymerase I	100	1.60e^-113^
gp19	1u3e_M	HNH homing endonuclease	99.92	2.90e^-25^
gp21	1exn_A	5'-exonuclease, 5'-nuclease	100	3.90e^-54^
gp22	1e7l_A	GP49, recombination endonuclease VII	100	8.10e^-42^
gp24	2gui_A	DNA polymerase III epsilon subunit	99.85	7.30e^-21^
gp27	1msw_D	DNA-directed RNA polymerase, bacteriophage T7 RNA	100	2.50e^-210^
gp29	4h89_A	GCN5-related N-acetyltransferase	96.86	0.002
gp32	3lj5_A	Portal protein, protein GP1	96.82	0.027
gp34	2xd8_A	GP10, T7-like capsid protein	100	1.30e^-48^
gp39	1qsa_A	Protein (soluble lytic transglycosylase SLT70)	99.15	6.20e^-11^
gp40	2ch7_A	Methyl-accepting chemotaxis protein	96.27	0.95
gp41	2kz6_A	Uncharacterized protein	99.67	1.70e^-16^
gp44	3cpe_A	Terminase, DNA packaging protein GP17	99.97	6.00e^-30^
gp47	3hde_A	Lysozyme	100	3.80e^-50^

HHpred hits with a probability >90% were included in the table.

A module commonly identified in other BCC phages that is notably absent from the JG068 genome is that for lysogeny. Similar to other obligately lytic phages, JG068 forms clear plaques and does not encode either an integrase or a repressor. Most other characterized members of the *Autographivirinae* are also virulent (although putative prophage elements described as being T7-like have been identified in *Xanthomonas axonopodis*, *Pseudomonas putida*, and *B. pseudomallei*) [39,40]. In order to verify experimentally that JG068 was not capable of lysogenizing BCC strains, we isolated JG068-insensitive K56-2 from a JG068/K56-2 lysate. To determine if these cells were insensitive due to either receptor mutation or superinfection immunity, we attempted to lyse these isolates using a second putatively virulent BCC-specific phage, KS12. Previous assays have shown that KS12 uses LPS as a receptor [Juárez Lara, unpublished data]. If JG068-insensitive K56-2 isolates are also insensitive to KS12 infection, they are likely LPS mutants, whereas if they are sensitive to KS12 infection, they are likely JG068 lysogens. Over 100 JG068-insensitive isolates were screened and all were found to be insensitive to both phages compared to wildtype K56-2. Twelve resistant colonies were PCR-screened using JG068-specific primers and each isolate was found to be amplification-negative. This evidence shows that resistance arises due to receptor mutation and not due to JG068 lysogeny.

**Figure 3:**
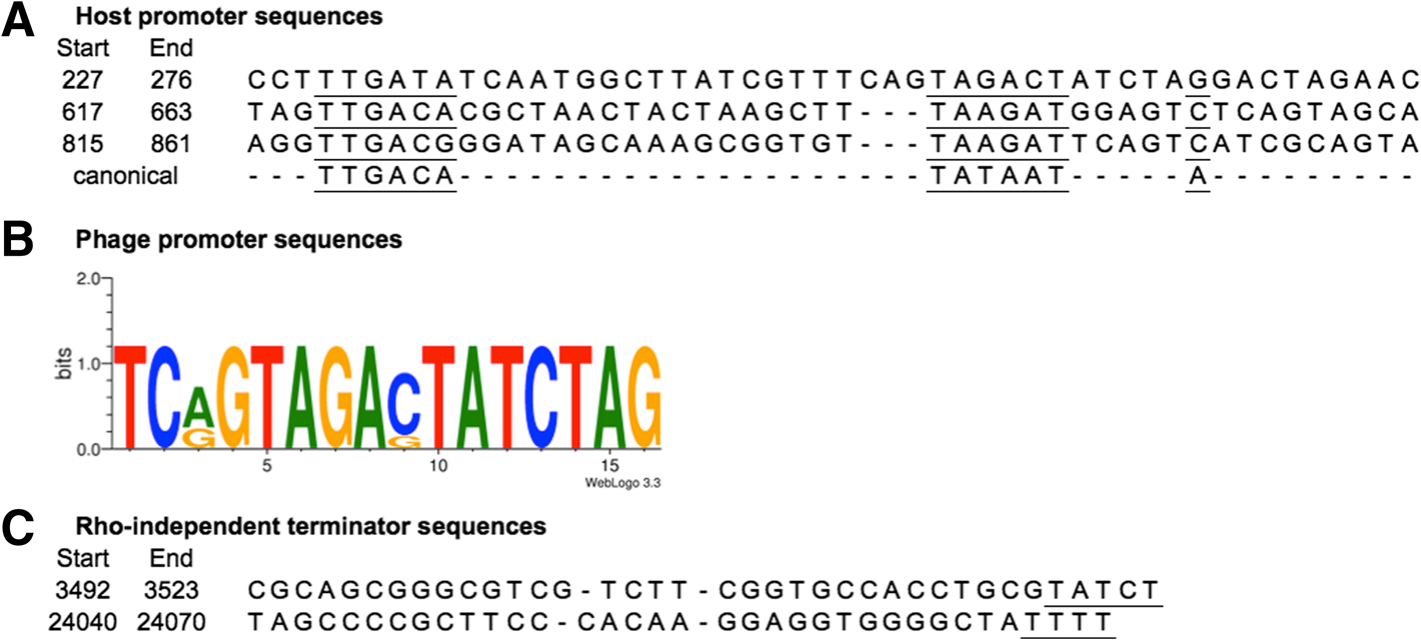
**Predicted promoter and terminator sequences in JG068. A**, putative host promoter sequences identified using Neural Network Promoter Prediction [50]. Predicted -35/-10 boxes and transcription start sites are underlined. A canonical prokaryotic promoter is shown below. **B**, putative phage promoter consensus sequence identified using PHIRE [51] and plotted using WebLogo 3.3 [52]. **C**, putative rho-independent terminator sequences identified using TransTermHP [53]. The central loop region is separated by dashes and the 3’ stretch of thymidine residues is underlined.

In *Autographivirinae*, transcription is first initiated from host promoters found at the far left-hand side of the genome, followed by initiation from phage RNA polymerase-specific promoters found throughout the genome [33,38]. JG068 promoters putatively recognized by BCC RNA polymerase were identified using Neural Network Promoter Prediction. Using a cutoff of 0.95 and limiting the results to sequences found intergenically, three promoters were identified in the first 650 bp downstream of the left DTR (Figure 2 and Figure 3A). Phage promoters lack the conserved structure observed in bacterial promoters with -10 and -35 regions and are instead described as short consensus sequences that vary among different phages [41]. PHIRE was used to identify a strongly conserved 16 bp consensus sequence (Figure 3B) found six times at five intergenic loci in the JG068 chromosome: overlapping the first host promoter sequence, upstream of the DNA polymerase gene *18* (2 promoters), upstream of the hypothetical protein gene *28* (downstream of the DNA-binding module), upstream of the capsid gene *34*, and upstream of the tail fiber gene *40* (Figure 2). Putative rho-independent terminators were identified using TransTermHP. Although multiple sequences were reported in the output, those shown in Figure 3C were chosen because they were intergenic (based on GeneMark predictions) and had a ΔG value of -10 kcal/mol or less. Similar to LIMEzero, the putative terminators are found upstream of the DNA-binding module and downstream of the capsid protein (Figure 2) [34].

### Module analysis

#### ***DNA-binding proteins***

One of the more notable aspects of the JG068 genome is its abundance of DNA-binding proteins. As discussed above, similar to T7 and ϕKMV, these genes are found centrally in the JG068 genome (Figure 2). In JG068 and other ϕKMV-like phages [32-34], the RNA polymerase gene is at the far right end of this module (upstream of the structural genes; Figure 2), whereas in T7 it is found close to the left end of the genome [38]. JG068 DNA-binding proteins include the primase gp12, helicase gp13, ligase gp17, DNA polymerase gp18, HNH endonuclease gp19, exonuclease gp21, endonuclease gp22, exonuclease gp24, and RNA polymerase gp27 (Table 2). The annotation of each of these proteins was based on BLASTP analysis and subsequently confirmed using HHpred (Tables 2 and 3).

One of the unifying features of the *Autographivirinae* is the presence of a single-subunit phage RNA polymerase gene [35]. Based on CGUG analysis, the only JG068 DNA-binding protein that shows similarity to those of T7 is this polymerase. These two proteins have 31% identity based on BLASTP analysis. In contrast, almost all of the JG068 DNA-binding proteins are similar to proteins in ϕKMV with 29-48% identity. Furthermore, these genes are found in the same order in these two phages.

The three components within this module that are dissimilar between ϕKMV and JG068 are the genes encoding the ligase, HNH endonuclease, and DNA binding protein. In ϕKMV, a ligase gene is found between the helicase and DNA polymerase (as in JG068), but the sequences of these genes are dissimilar between the two phages [32]. Several HNH homing endonuclease genes have been identified in T7 [38], but only one is found in JG068 and none have been identified in ϕKMV. Although a DNA binding protein gene was identified upstream of the helicase gene in ϕKMV [32], such a gene was not identified in the JG068 annotation.

The position of the promoters driving the expression of genes in this module also differs between these phages. In ϕKMV, promoters upstream of these genes include four host promoters and two phage promoters (one between the DNA binding protein and primase genes and one between the DNA polymerase and endonuclease genes) [32]. In contrast, whereas the host promoters for JG068 are found at a similar locus as in ϕKMV, the phage promoters either overlap the host promoters or are found between the ligase (*17*) and DNA polymerase (*18*) genes (Figure 2). Based on this arrangement, all of the ϕKMV genes in this module could be expressed from internal phage promoters (except for the gene encoding the DNA binding protein), whereas the primase (*12*), helicase (*13*), and ligase (*17*) genes of JG068 would be expressed from either a host promoter or the nested phage promoter on the far left end (Figure 2).

#### ***Morphogenesis proteins***

The structural proteins of ϕKMV, LKA1, and LKD16 have been identified using mass spectrometry of proteins from purified virions [33,42]. Based on CGUG analysis, JG068 encodes proteins similar to the majority of these structural proteins. The putative structural genes *32*–*40* (excluding *33*, discussed below) are contained in a single 17 kbp module (Figure 2). In a CGUG comparison of ϕKMV and JG068, the head-tail connector (JG068 gp32), capsid protein (gp34), tail tubular protein A (gp35) and B (gp36), internal virion proteins (gp38 and gp39), and tail fiber protein (gp40) are all similar and each of these proteins was shown to be structural in ϕKMV virions [42]. In addition, when JG068 and LKA1 are compared using CGUG, a third internal virion protein (gp37) is identified as similar that is structural in LKA1 [33]. ϕKMV, LKA1, and LKD16 virions also contain some proteins not encoded similarly by JG068. The genes encoding these structural proteins are found either upstream of the head-tail connector gene or downstream of the tail fiber gene and have uncharacterized functions or are putatively involved in adsorption [33,42]. Based on our genome annotation, JG068 does not encode any additional unique structural proteins.

Additional head morphogenesis/DNA packaging and tail morphogenesis proteins are encoded by JG068 that are not predicted to be associated with the mature virion. Although the scaffolding protein gp33 is encoded within the structural module discussed above, it is likely to function in capsid assembly but not to be part of the mature virion based on ϕKMV, LKA1, and LKD16 structural protein analysis [33,42]. Dissimilar to many other phages (but similar to others in the ϕKMV-like genus) [32-34], the DNA packaging genes *43* and *44* are found far downstream of the head morphogenesis genes (Figure 2). Gp33, gp43, and gp44 are all similar to proteins of ϕKMV based on CGUG analysis. The only putative morphogenesis protein that is unique to JG068 and not shared by ϕKMV or LKA1 is the putative tail fiber assembly protein gp41. Although HHpred analysis was uninformative (as the high probability matches to gp41 lack characterized functions; Table 3), this protein shows similarity in BLASTP analysis to tail fiber assembly proteins of *Cupriavidus necator* N-1, *Dickeya dadantii* 3937, and *Idiomarina xiamenensis* 10-D-4.

#### ***Lysis proteins***

The lysis proteins of JG068, encoded on the far right end of the genome, include a putative pinholin, signal-arrest-release (SAR) endolysin, Rz, and Rz1. Although the lysis genes in most phages (including ϕKMV) tend to be arranged in a single block with the holin gene followed by the endolysin, Rz, and Rz1 genes [43], this order is not maintained in JG068. Here, the putative pinholin gene *42* is found upstream of the SAR endolysin (*47*), Rz (*48*), and Rz1 (*49*) genes, separated by genes encoding two DNA packaging proteins and two hypothetical proteins (Figure 2). Within the *Autographivirinae* subfamily, this gene arrangement is also observed in SP6 [44].

Although only two of the four JG068 lysis proteins are similar to those of ϕKMV based on CGUG analysis (the SAR endolysin gp47 and Rz gp48), these two phages are likely to use similar lysis mechanisms. The gp42 pinholin has analogous features to that of ϕKMV: ~60 amino acids in length (60 for JG068 and 66 for ϕKMV), two transmembrane domains (as predicted by TMHMM analysis), and positively-charged arginine and lysine residues at the C-terminus [43]. The gp48 Rz inner membrane protein has a single N-terminal transmembrane domain based on TMHMM analysis. Gene *49*, encoding the Rz1 outer membrane lipoprotein, overlaps with gene *48* in the +1 reading frame and extends downstream of the *48* stop codon by 148 base pairs (Figure 2). LipoP analysis predicts a signal peptidase II cleavage site in gp49 between residues 18 (alanine) and 19 (cysteine).

The SAR endolysin of JG068 has a very similar organization to that of ϕKMV. SignalP 3.0 predicts an N-terminal signal sequence probability of 0.97 for gp47 (later versions of SignalP do not make the same prediction, likely because the C-region is absent). The gp47 N-terminus contains two signal sequence regions followed by three putative catalytic residues of the lysozyme domain. Residues 1–7, including a positively-charged histidine, lysine, and arginine, make up the N-region. Residues 8–25 make up the hydrophobic glycine- and alanine-rich H-region, also predicted to be a transmembrane domain by OCTOPUS analysis. Using CD Search and BLASTP alignment with the ϕKMV SAR endolysin, the putative lysozyme catalytic residues were identified as 27E, 36D, and 45T, all found immediately downstream of the H-region. As in ϕKMV, because the C-region is absent, the protein will not be cleaved by a signal peptidase upon secretion and will instead remain associated with the inner membrane until release into the periplasm [43].

**Figure 4:**
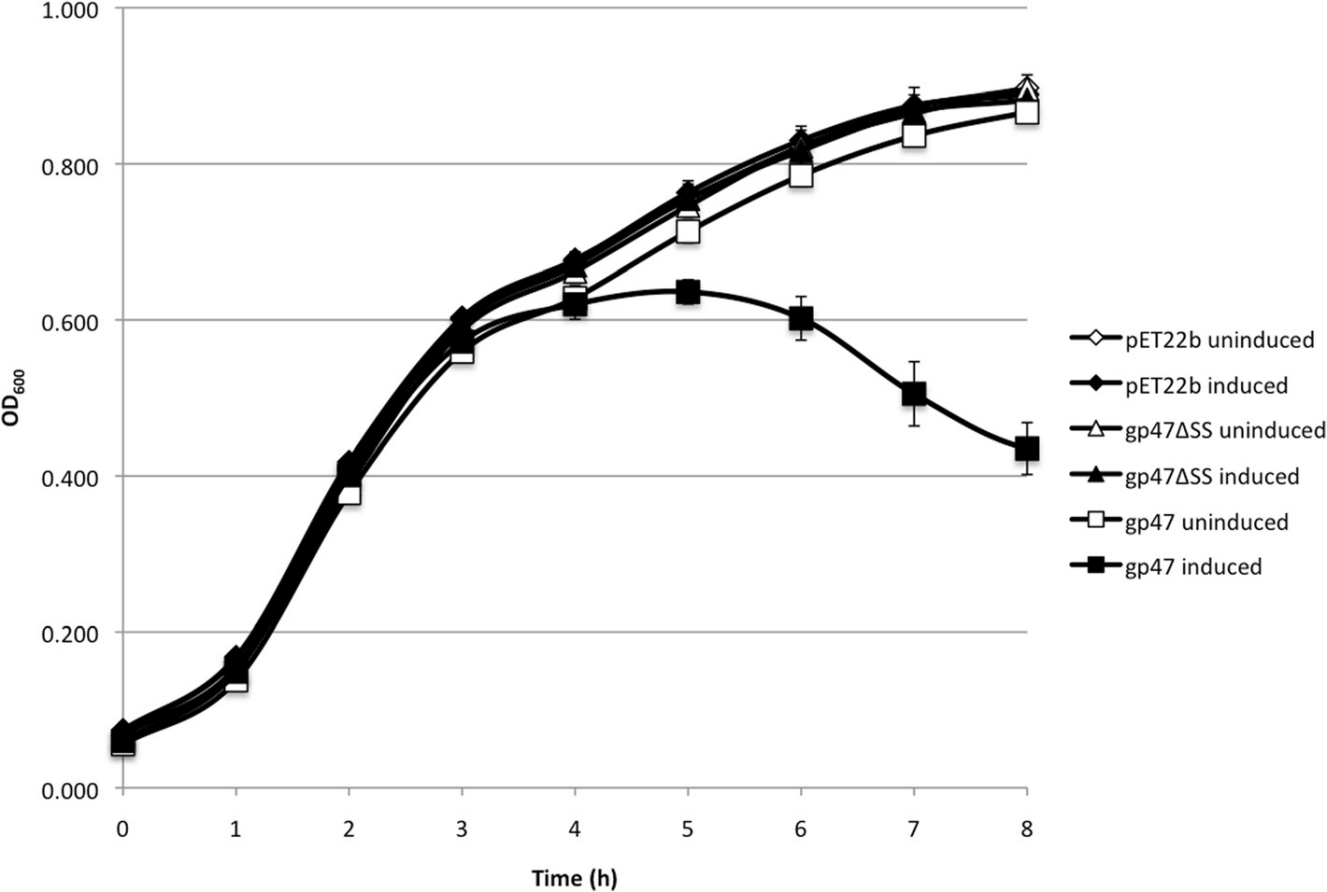
**Signal sequence-dependent lytic activity of the gp47 SAR endolysin expressed in *E. coli *BL21(DE3)pLysS.** Cells carrying pET22b or expressing either gp47 or gp47 lacking the putative signal sequence (gp47ΔSS) were subcultured and incubated with shaking at 37°C. Cells were induced with 1 mM IPTG (final concentration) at 3 h (OD_600_ ~ 0.6).

Using inducible expression of the ϕKMV SAR endolysin in *E. coli*, it was shown that this protein could decrease culture absorbance in the absence of a pinholin, but only if the N-terminal signal sequence of the endolysin was present [43]. We performed a similar experiment using the JG068 SAR endolysin to further characterize the export mechanism and biological activity of this protein. To allow for tightly controlled inducible expression, we cloned gene *47* into pET22b (with [gp47] or without [gp47ΔSS] the putative signal sequence in the first 25 residues) and transformed these plasmids into *E. coli* BL21(DE3)pLysS. When these cells (or a pET22b blank control) are subcultured, their growth rates prior to IPTG induction are similar based on optical density measurements at 600 nm (OD_600_; up to 3 h in Figure 4). However, following induction, expression of gp47 is lethal to the cells as the OD_600_ decreases from ~0.6 at 3 hours to ~0.45 at 8 hours (black squares, Figure 4). A very different trend is observed for the blank control, gp47ΔSS, and uninduced gp47 strains, where the OD_600_ increases from ~0.6 at 3 hours to ~0.9 at 8 hours, double that of the induced gp47 OD_600_ (Figure 4). As the lytic activity is dependent upon expression of not only the lysozyme domain but also the signal sequence, we can conclude that JG068 gp47 acts as a typical SAR endolysin in Gram-negative bacteria. While the classical endolysins of Bcep781/Bcep43 and BcepC6B have been functionally characterized [15,45], this is the first experimental evidence for SAR endolysin activity in a BCC phage. As these proteins have also been identified in the Bcep22-like viruses [25,46], similar experiments may be used to confirm the activity of SAR endolysins in these phages as well.

### *In vivo* activity

**Figure 5:**
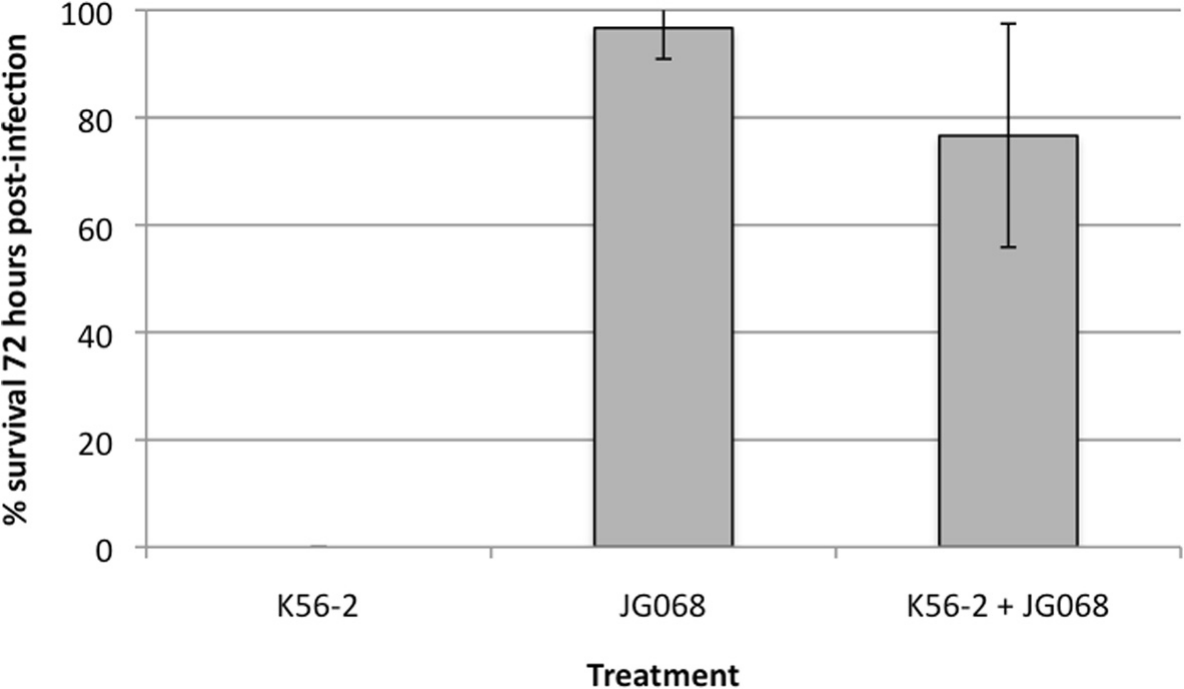
***In vivo *activity of JG068 against *B. cenocepacia *K56-2.***G. mellonella* larvae were infected with K56-2 and treated with endotoxin-removed JG068 at a multiplicity of infection (MOI) of 350. Survival was assessed 72 hours post-infection.

The *Galleria mellonella* (greater wax moth) larvae model is commonly used to assess both strain virulence and phage therapy efficacy for members of the BCC, particularly *B. cenocepacia*[10,11,18]. Although this model is less complex than a mammalian system, it shows positive correlation with both mouse and rat models of infection [18]. To determine if JG068 possesses lytic activity against *B. cenocepacia in vivo*, we infected *G. mellonella* larvae with 3.7 × 10^3^ colony forming units (CFU) of K56-2 and treated with endotoxin-removed JG068 at a multiplicity of infection (MOI) of 350 (1.3 × 10^6^ plaque forming units [PFU]). For infected, untreated controls, no larvae survived after 72 hours (Figure 5, left). Those larvae that received only JG068 remained healthy over the course of the experiment (Figure 5, centre), indicating that phage treatment produced no harmful effects. For infected, treated larvae, JG068 administration significantly increased survival to an average of 77% (from 0% in untreated controls) (Figure 5, right). In a previous study, the putatively virulent phage KS12 was the most effective for immediate treatment of K56-2 infection in the *G. mellonella* model, resulting in 93 ± 12% survival (MOI = 5000) or 57 ± 6% survival (MOI = 500) [10]. Based on the *G. mellonella* data presented in Figure 5, JG068 is almost as effective as a significantly larger dose of KS12, indicating that JG068 is highly active *in vivo*.

## Conclusions

One of the greatest challenges in BCC phage therapy development is the identification of phages with a broad, clinically relevant host range that are free of virulence genes, obligately lytic, and active *in vivo*. Our characterization of the podovirus JG068 indicates that it is a rare example of a BCC phage that satisfies each of these requirements. JG068 infects strains of *B. multivorans*, *B. cenocepacia* (interacting with the LPS core as a receptor in K56-2 and PC184), *B. stabilis*, and *B. dolosa*. It is the first BCC virus to be identified as a member of the *Autographivirinae* subfamily and ϕKMV-like phages genus. The 41,604 bp genome sequence has a structure similar to that of other *Autographivirinae* chromosomes, with DTRs and genes encoding DNA-binding, morphogenesis, and lysis proteins expressed in the forward direction from phage and host promoters. JG068 lacks virulence genes, is obligately lytic, and encodes a functional SAR endolysin, the first to be experimentally verified for the BCC. Administration of JG068 to *B. cenocepacia* K56-2-infected *G. mellonella* larvae significantly decreases larval mortality, providing the first evidence that sequenced, obligately lytic BCC phages are active against *B. cenocepacia in vivo*. Given these characteristics, further study is warranted regarding the development of JG068 into an active antimicrobial for use in CF patients.

## Methods

### Bacterial strains and culture conditions

BCC strains were grown aerobically at 30°C overnight on half-strength Luria-Bertani (½ LB) solid agar or agarose plates or in ½ LB broth. Strains used for host range testing and phage propagation are members of the original and updated BCC experimental strain panels [19,20]. K56-2 LPS mutants [27,28] were grown at 30°C on ½ LB plates or in ½ LB broth containing 100 mg/l trimethoprim. *E. coli* DH5α transformed with pUC19 or pET22b was grown at 37°C on LB plates containing 100 mg/l ampicillin. *E. coli* BL21(DE3)pLysS was grown at 37°C in LB broth containing 25 mg/l chloramphenicol or, if transformed with pET22b, 25 mg/l chloramphenicol and 100 mg/l ampicillin.

### Phage propagation, host range analysis, lysogeny screen, and electron microscopy

JG068 was isolated from a sewage plant in Steinhof near Braunschweig in Germany following an enrichment protocol previously described [47]. For propagation of JG068, 100 μl high-titre phage stock and 100 μl BCC liquid culture were combined and incubated 20 minutes at room temperature, mixed with 3 ml 0.7% ½ LB agar or 0.6% ½ LB agarose, overlaid on a ½ LB plate, and incubated at 30°C overnight. High-titre stocks were made in modified suspension medium (modified SM; 50 mM Tris–HCl [pH 7.5], 100 mM NaCl, 10 mM MgSO_4_). Phage plates were overlaid with 3 ml modified SM and incubated >1 h at room temperature on a platform rocker. The supernatant was recovered, pelleted by centrifugation for 2 min at 10,000 × g, filter-sterilized using a Millex-HA 0.45 μm syringe-driven filter unit (Millipore, Billerica, MA), and stored at 4°C.

Spot testing was used for preliminary host range analysis. Soft-agar overlays containing 100 μl BCC liquid culture were allowed to solidify for ≥10 minutes at room temperature, spotted with 10 μl drops of high-titer JG068 stock, and assayed for clearing and/or plaque formation after incubation at 30°C overnight. Strains that showed phage susceptibility in this assay were re-tested in soft-agar overlays containing both the strain and JG068. The host ranges for LPS mutants of K56-2 [27,28] and PC184 [Abdu, unpublished data] were tested similarly. Efficiency of plating (EOP) values were determined as previously described [46].

To assay for JG068-lysogenized K56-2, JG068 and K56-2 were plated in agar overlays at a multiplicity of infection of 10^0^ and incubated at 30°C overnight. To recover surviving cells, plates were overlaid with 3 ml sterile water and incubated 1 h at room temperature on a platform rocker. Cells were pelleted by centrifugation for 2 min at 10,000 x g, washed and resuspended in sterile water, and plated to obtain isolated colonies. Colonies were struck out onto ½ LB plates and spotted with separate 10 μl drops of high-titer JG068 stock and high-titer KS12 stock. Isolates were scored as sensitive or insensitive compared to a sensitive wildtype K56-2 control that was lysed by both phage stocks. A subset of resistant isolates was replated and PCR-screened for lysogeny using JG068-specific primers (9F: GAACATCGGTAACGTCGTCAAGG; 9R: GGCGTGACGAACAGCTTGGC). TopTaq DNA polymerase and buffers (Qiagen, Hilden, Germany) were used according to the manufacturer’s instructions (template: 1 μl overnight culture incubated 5 min at 100°C).

For electron microscopy, phage stocks were prepared as described above with the following modifications: agarose plates and soft agarose were used for overlays, sterile water was used in place of modified SM, and a 0.22 μm filter was used for syringe-driven filtration. A carbon-coated copper grid was incubated with lysate for 5 min and stained with 2% phosphotungstic acid for 30 s. Transmission electron micrographs were captured using a Philips/FEI (Morgagni) transmission electron microscope with charge-coupled device camera at 80 kV (University of Alberta Department of Biological Sciences Advanced Microscopy Facility). The capsid diameter (mean ± standard deviation) was calculated using Microsoft Excel based on measurements from nine individual virions.

### Phage DNA isolation, RFLP analysis, and sequencing

Phage DNA was isolated using a modified version of a Wizard DNA Clean-Up protocol [48]. Ten milliliters of filter-sterilized JG068 lysate (propagated using K56-2 on agarose medium) was treated with 10 μl DNase I (Thermo Scientific, Waltham, MA), 100 μl 100x DNase I buffer (1 M Tris–HCl, 0.25 M MgCl_2_, 10 mM CaCl_2_), and 6 μl RNase (Thermo Scientific) and incubated 1 h at 37°C to degrade the bacterial nucleic acids. Four hundred microliters of 0.5 M EDTA and 25 μl of 20 mg/ml proteinase K (Applied Biosystems, Carlsbad, CA) were added and incubated 1 h at 55°C to inactivate DNase I. After cooling to room temperature, the lysate was added to 8.4 g of guanidine thiocyanate and briefly mixed. One milliliter of warmed, resuspended Wizard DNA Clean-Up Resin (Promega Corporation, Madison, WI) was added to the mixture and mixed by inverting for 15 minutes to allow for release of the phage DNA and binding to the resin. The mixture was pelleted by centrifugation at room temperature for 10 min at 5,000 x g and the supernatant was drawn off until ~5 ml remained. This mixture was resuspended by swirling, transferred into an empty syringe barrel attached to a Wizard Minicolumn (Promega Corporation), and pushed into the column. The column was then washed with 2 ml 80% isopropanol and dried by centrifugation for 2 min at 10,000 x g. JG068 DNA was eluted from the column following addition of 100 μl of 80°C nuclease-free water (Integrated DNA Technologies, Coralville, IA), incubation for 1 min, and centrifugation for ≥20 s at 10,000 x g.

Phage and plasmid DNA were quantified using a NanoDrop 2000c spectrophotometer (Thermo Scientific). For shotgun cloning, JG068 EcoRI fragments were purified using a GENECLEAN III kit (MP Biomedicals, Santa Ana, CA), ligated into pUC19, and transformed into *E. coli* DH5α (Invitrogen, Carlsbad, CA). Plasmids were purified using the GeneJET Plasmid Miniprep kit (Thermo Scientific) and sequenced using a 3730 DNA Analyzer (Applied Biosystems) by the University of Alberta Department of Biological Sciences Molecular Biology Service Unit (MBSU). Sequences were edited and assembled using Geneious [49].

The complete JG068 genome sequence was determined with the assistance of the MBSU using an Ion Torrent PGM (Applied Biosystems) and assembled using SeqMan NGen (DNASTAR, Madison, WI). Ambiguous regions in the assembly were resequenced using Sanger sequencing from EcoRI clones (for internal fragments) or PCR products (for terminal fragments). Chromosomal termini were identified using primers within the left direct terminal repeat (DTR), extending to the left end (IL: CAACCCTGTACAGCCGACCC), outside of the left DTR, extending to the left end (OL: CCTTGCTCTATCTACCATGTTCCGC), within the right DTR, extending to the right end (IR: TGTGGATAGGGCGAAGTCTGAAGC), and outside of the right DTR, extending to the right end (OR: CTCCGACGAAGCATCCGC). Primers were used to directly sequence the JG068 DNA and sequence alignment was used to identify the loci where sequence intensity dropped by 50% (for primers IL and IR) or 100% (for primers OL and OR). The JG068 sequence has been deposited in GenBank [GenBank: KC853746].

### Bioinformatics analysis

Open reading frames (ORFs) were identified using GeneMark.hmm for prokaryotes [54] and annotated using BLASTP [55], HHpred [56], and CD-Search [57]. Gene *49*, which has a start codon within the last third of gene *48*, was identified using ORF Finder [58]. The replication origin and terminus were predicted using a GC-skew plot generated by GenSkew [59]. Genome restriction profiles were predicted using NEBcutter [60]. Whole genome comparisons were performed using CoreGenesUniqueGenes (CGUG) with a cutoff score of 75 [35,37]. Screening for bacterial toxins was performed using BTXpred [61]. Bacterial promoters, phage promoters, and rho-independent terminators were identified using prokaryotic Neural Network Promoter Prediction with a cutoff of 0.95 [50], PHIRE [51], and TransTermHP [53] on the PePPER server [62], respectively. Sequence logos were constructed using WebLogo [52]. Transmembrane domains were predicted using TMHMM [63] and OCTOPUS [64]. Lysis protein signal sequences were identified using SignalP 3.0 [65] and LipoP [66].

### SAR endolysin expression

To assess the activity of the gp47 SAR endolysin, gene *47* including the signal sequence (bp 40,232 – 40,753; NdeI-SS-F: ATAATAACATATGCACCCCATCGTCAAGCGAG; HindIII-R: AAAAAGCTTCTATCGCCGGATACCAGCAACG) or excluding the signal sequence (bp 40,307 – 40,753; NdeI-ΔSS-F: TAATAACATATGGACGAGGGTATCCGGAACGTC; HindIII-R: as above) was PCR amplified using KAPA HiFi HotStart ReadyMix (Kapa Biosystems, Woburn, MA) and ligated in-frame into pET22b. These constructs were separately transformed into *E. coli* DH5α, sequenced to verify that the inserts were correct, and transformed into *E. coli* BL21(DE3)pLysS using a standard protocol [67]. A pET22b blank control plasmid was isolated from *E. coli* MC4100 and transformed similarly into BL21(DE3)pLysS. To assay endolysin activity in *E. coli*, 5 ml liquid cultures of the three strains (in triplicate) were each subcultured into six wells of a 96 well plate by adding 10 μl culture to 190 μl LB broth containing 25 mg/l chloramphenicol and 100 mg/l ampicillin. The plate was then covered with plastic wrap and incubated with shaking at 37°C. Optical density measurements at 600 nm (OD_600_) were measured at 1 h intervals with a Wallac 1420 VICTOR^2^ multilabel counter (PerkinElmer, Waltham, MA). At 3 h (OD_600_ ~ 0.6), half of the samples were induced with IPTG (1 mM final concentration) and optical density measurements continued at 1 h intervals up to 8 h. Averages and standard deviations were calculated using Microsoft Excel.

### *Galleria mellonella* assays

*G. mellonella* infection and treatment assays were performed as described previously [10] with modifications. One milliliter of a 16 h K56-2 overnight culture was pelleted by centrifugation for 2 min at 10,000 × g, resuspended in 1 ml MgSO_4_-ampicillin solution (10 mM MgSO_4_, 1.2 mg/ml ampicillin), and diluted in this solution to 1:10^4^. High-titre JG068 lysate was passaged through a Detoxi-Gel Endotoxin Removing Column (Thermo Scientific) and supplemented with ampicillin. Larvae (RECORP Inc., Georgetown, ON) were stored at 4°C and warmed to room temperature prior to injection with a 250 μl syringe with repeating dispenser (Hamilton Company, Reno, NV). In the left hindmost proleg, 5 μl of the diluted K56-2 suspension (3.7 × 10^3^ CFU/5 μl) was injected into ten infected/treated larvae and ten infected/untreated larvae and 5 μl of MgSO_4_-ampicillin solution was injected into ten uninfected/treated larvae. In the adjacent left proleg, 5 μl of the JG068 lysate (1.3 × 10^6^ PFU/5 μl) was injected into ten infected/treated larvae and ten uninfected/treated larvae and 5 μl of MgSO_4_-ampicillin solution was injected into ten infected/untreated larvae. Larvae were placed into petri dishes, incubated aerobically at 30°C for 72 h, and scored for survival. Experiments were repeated in triplicate. Averages and standard deviations were calculated using Microsoft Excel.

## Competing interests

The authors declare that they have no competing interests.

## Authors’ contributions

KHL and AHA carried out the electron microscopy and sequenced and assembled the JG068 genome. KHL annotated the genome, performed bioinformatics analyses, and drafted the manuscript. AHA determined the BCC and LPS mutant host ranges, performed the SAR endolysin and *G. mellonella* experiments, and edited the manuscript. MS isolated JG068. JJD planned, supervised, and coordinated the study and edited the manuscript. All authors read and approved the final manuscript.

## References

[B1] Falagas ME Bliziotis IA Kasiakou SK Samonis G Athanassopoulou P Michalopoulos A Outcome of infections due to pandrug-resistant (PDR) Gram-negative bacteria BMC Infect Dis 2005 5 24 10.1186/1471-2334-5-24 15819983 PMC1087841

[B2] Merril CR Scholl D Adhya SL The prospect for bacteriophage therapy in Western medicine Nat Rev Drug Discov 2003 2 489 497 10.1038/nrd1111 12776223

[B3] Bruttin A Brüssow H Human volunteers receiving *Escherichia coli* phage T4 orally: a safety test of phage therapy Antimicrob Agents Chemother 2005 49 2874 2878 10.1128/AAC.49.7.2874-2878.2005 15980363 PMC1168693

[B4] Merabishvili M Pirnay J-P Verbeken G Chanishvili N Tediashvili M Lashkhi N Glonti T Krylov V Mast J Van Parys L Lavigne R Volckaert G Mattheus W Verween G De Corte P Rose T Jennes S Zizi M De Vos D Vaneechoutte M Quality-controlled small-scale production of a well-defined bacteriophage cocktail for use in human clinical trials PLoS ONE 2009 4 e4944 10.1371/journal.pone.0004944 19300511 PMC2654153

[B5] Rhoads DD Wolcott RD Kuskowski MA Wolcott BM Ward LS Sulakvelidze A Bacteriophage therapy of venous leg ulcers in humans: results of a phase I safety trial J Wound Care 2009 18 237 243 19661847 10.12968/jowc.2009.18.6.42801

[B6] Wright A Hawkins CH Änggård EE Harper DR A controlled clinical trial of a therapeutic bacteriophage preparation in chronic otitis due to antibiotic-resistant *Pseudomonas aeruginosa*; A preliminary report of efficacy Clin Otolaryngol 2009 34 349 357 10.1111/j.1749-4486.2009.01973.x 19673983

[B7] Mahenthiralingam E Urban TA Goldberg JB The multifarious, multireplicon *Burkholderia cepacia* complex Nat Rev Microbiol 2005 3 144 156 10.1038/nrmicro1085 15643431

[B8] Zhou J Chen Y Tabibi S Alba L Garber E Saiman L Antimicrobial susceptibility and synergy studies of *Burkholderia cepacia* complex isolated from patients with cystic fibrosis Antimicrob Agents Chemother 2007 51 1085 1088 10.1128/AAC.00954-06 17158942 PMC1803131

[B9] Adachi N Tsukamoto S Inoue Y Azegami K Control of bacterial seedling rot and seedling blight of rice by bacteriophage Plant Dis 2012 96 1033 1036 10.1094/PDIS-03-11-0232-RE 30727215

[B10] Seed KD Dennis JJ Experimental bacteriophage therapy increases survival of *Galleria mellonella* larvae infected with clinically relevant strains of the *Burkholderia cepacia* complex Antimicrob Agents Chemother 2009 53 2205 2208 10.1128/AAC.01166-08 19223640 PMC2681512

[B11] Lynch KH Seed KD Stothard P Dennis JJ Inactivation of *Burkholderia cepacia* complex phage KS9 gp41 identifies the phage repressor and generates lytic virions J Virol 2010 84 1276 1288 10.1128/JVI.01843-09 19939932 PMC2812329

[B12] Carmody LA Gill JJ Summer EJ Sajjan US Gonzalez CF Young RF LiPuma JJ Efficacy of bacteriophage therapy in a model of *Burkholderia cenocepacia* pulmonary infection J Infect Dis 2010 201 264 271 10.1086/649227 20001604 PMC2814432

[B13] Lynch KH Dennis JJ Cangene gold medal award lecture - Genomic analysis and modification of *Burkholderia cepacia* complex bacteriophages Can J Microbiol 2012 58 221 235 10.1139/w11-135 22339239

[B14] Lynch KH Dennis JJ Coenye T, Mahenthiralingam E Genomics of *Burkholderia* phages Burkholderia: From Genomes to Function Hethersett: Horizon Scientific Press In press

[B15] Summer EJ Gonzalez CF Bomer M Carlile T Embry A Kucherka AM Lee J Mebane L Morrison WC Mark L King MD LiPuma JJ Vidaver AK Young R Divergence and mosaicism among virulent soil phages of the *Burkholderia cepacia* complex J Bacteriol 2006 188 255 268 10.1128/JB.188.1.255-268.2006 16352842 PMC1317576

[B16] Robinson SM Hall J Das A Rosenbloom A Gonzalez C Young R Summer EJ M-015. Genomic analysis of virulent soil phages of Burkholderia http://ieg.ou.edu/asm2006/data/papers/M_015.htm

[B17] Pirnay J-P Verbeken G Rose T Jennes S Zizi M Huys I Lavigne R Merabishvili M Vaneechoutte M Buckling A De Vos D Introducing yesterday's phage therapy in today's medicine Future Virol 2012 7 379 390 10.2217/fvl.12.24

[B18] Seed KD Dennis JJ Development of *Galleria mellonella* as an alternative infection model for the *Burkholderia cepacia* complex Infect Immun 2008 76 1267 1275 10.1128/IAI.01249-07 18195031 PMC2258804

[B19] Mahenthiralingam E Coenye T Chung JW Speert DP Govan JRW Taylor P Vandamme P Diagnostically and experimentally useful panel of strains from the *Burkholderia cepacia* complex J Clin Microbiol 2000 38 910 913 10655415 10.1128/jcm.38.2.910-913.2000 PMC86244

[B20] Coenye T Vandamme P LiPuma JJ Govan JRW Mahenthiralingam E Updated version of the *Burkholderia cepacia* complex experimental strain panel J Clin Microbiol 2003 41 2797 2798 10.1128/JCM.41.6.2797-2798.2003 12791937 PMC156479

[B21] Broad Institute Burkholderia dolosa databasehttp://www.broadinstitute.org/annotation/genome/burkholderia_dolosa

[B22] Seed KD Dennis JJ Isolation and characterization of bacteriophages of the *Burkholderia cepacia* complex FEMS Microbiol Lett 2005 251 273 280 10.1016/j.femsle.2005.08.011 16140471

[B23] Goudie AD Lynch KH Seed KD Stothard P Shrivastava S Wishart DS Dennis JJ Genomic sequence and activity of KS10, a transposable phage of the *Burkholderia cepacia* complex BMC Genomics 2008 9 615 10.1186/1471-2164-9-615 19094239 PMC2628397

[B24] Lynch KH Stothard P Dennis JJ Genomic analysis and relatedness of P2-like phages of the *Burkholderia cepacia* complex BMC Genomics 2010 11 599 10.1186/1471-2164-11-599 20973964 PMC3091744

[B25] Lynch KH Stothard P Dennis JJ Characterization of DC1, a broad-host-range Bcep22-like podovirus Appl Environ Microbiol 2012 78 889 891 10.1128/AEM.07097-11 22139000 PMC3264122

[B26] Lynch KH Stothard P Dennis JJ Comparative analysis of two phenotypically-similar but genomically-distinct *Burkholderia cenocepacia*-specific bacteriophages BMC Genomics 2012 13 223 10.1186/1471-2164-13-223 22676492 PMC3483164

[B27] Loutet SA Flannagan RS Kooi C Sokol PA Valvano MA A complete lipopolysaccharide inner core oligosaccharide is required for resistance of *Burkholderia cenocepacia* to antimicrobial peptides and bacterial survival in vivo J Bacteriol 2006 188 2073 2080 10.1128/JB.188.6.2073-2080.2006 16513737 PMC1428139

[B28] Ortega X Silipo A Saldfas MS Bates CC Molinaro A Valvano MA Biosynthesis and structure of the *Burkholderia cenocepacia* K56-2 lipopolysaccharide core oligosaccharide: Truncation of the core oligosaccharide leads to increased binding and sensitvity to polymyxin B J Biol Chem 2009 284 21738 21751 10.1074/jbc.M109.008532 19525227 PMC2755896

[B29] Ackermann H-W Frequency of morphological phage descriptions in the year 2000 Arch Virol 2001 146 843 857 10.1007/s007050170120 11448025

[B30] Casjens SR Gilcrease EB Determining DNA packaging strategy by analysis of the termini of the chromosomes in tailed-bacteriophage virions Methods Mol Biol 2009 502 91 111 10.1007/978-1-60327-565-1_7 19082553 PMC3082370

[B31] Summer EJ Preparation of a phage DNA fragment library for whole genome shotgun sequencing Methods Mol Biol 2009 502 27 46 10.1007/978-1-60327-565-1_4 19082550

[B32] Lavigne R Burkal'tseva MV Robben J Sykilinda NN Kurochkina LP Grymonprez B Jonckx B Krylov VN Mesyanzhinov VV Volckaert G The genome of bacteriophage ϕKMV, a T7-like virus infecting *Pseudomonas aeruginosa* Virology 2003 312 49 59 10.1016/S0042-6822(03)00123-5 12890620

[B33] Ceyssens P-J Lavigne R Mattheus W Chibeu A Hertveldt K Mast J Robben J Volckaert G Genomic analysis of *Pseudomonas aeruginosa* phages LKD16 and LKA1: establishment of the ϕKMV subgroup within the T7 supergroup J Bacteriol 2006 188 6924 6931 10.1128/JB.00831-06 16980495 PMC1595506

[B34] Adriaenssens EM Ceyssens P-J Dunon V Ackermann H-W Van Vaerenbergh J Maes M De Proft M Lavigne R Bacteriophages LIMElight and LIMEzero of *Pantoea agglomerans*, belonging to the "ϕKMV-like viruses" Appl Environ Microbiol 2011 77 3443 3450 10.1128/AEM.00128-11 21421778 PMC3126476

[B35] Lavigne R Seto D Mahadevan P Ackermann H-W Kropinski AM Unifying classical and molecular taxonomic classification: analysis of the *Podoviridae* using BLASTP-based tools Res Microbiol 2008 159 406 414 10.1016/j.resmic.2008.03.005 18555669

[B36] Abbasifar R Kropinski AM Sabour PM Ackermann H-W Alanis Villa A Abbasifar A Griffiths MW The genome of *Cronobacter sakazakii* bacteriophage vB_CsaP_GAP227 suggests a new genus within the *Autographivirinae* Genome Announc 2013 1 e00122-12 10.1128/genomeA.00122-12 23409275 PMC3569369

[B37] Mahadevan P King JF Seto D CGUG: in silico proteome and genome parsing tool for the determination of "core" and unique genes in the analysis of genomes up to ca. 1.9 Mb BMC Res Notes 2009 2 168 10.1186/1756-0500-2-168 19706165 PMC2738686

[B38] Molineux IJ Calendar R The T7 Group The Bacteriophages 2006 2 New York: Oxford University Press 277 301

[B39] Casjens S Prophages and bacterial genomics: What have we learned so far? Mol Microbiol 2003 49 277 300 10.1046/j.1365-2958.2003.03580.x 12886937

[B40] Kawasaki T Shimizu M Satsuma H Fujiwara A Fujie M Usami S Yamada T Genomic characterization of *Ralstonia solanacearum* phage ϕRSB1, a T7-like wide-host-range phage J Bacteriol 2009 191 422 427 10.1128/JB.01263-08 18952798 PMC2612426

[B41] Lavigne R Villegas A Kropinksi AM *In silico* characterization of DNA motifs with particular reference to promoters and terminators Methods Mol Biol 2009 502 113 129 10.1007/978-1-60327-565-1_8 19082554

[B42] Lavigne R Noben JP Hertveldt K Ceyssens P-J Briers Y Dumont D Roucourt B Krylov VN Mesyanzhinov VV Robben J Volckaert G The structural proteome of *Pseudomonas aeruginosa* bacteriophage ϕKMV Microbiology 2006 152 529 534 10.1099/mic.0.28431-0 16436440

[B43] Briers Y Peeters LM Volckaert G Lavigne R The lysis cassette of bacteriophage ϕKMV encodes a signal-arrest-release endolysin and a pinholin Bacteriophage 2011 1 25 30 10.4161/bact.1.1.14868 21687532 PMC3109451

[B44] Dobbins AT George M Jr Basham DA Ford ME Houtz JM Pedulla ML Lawrence JG Hatfull GF Hendrix RW Complete genomic sequence of the virulent *Salmonella* bacteriophage SP6 J Bacteriol 2004 186 1933 1944 10.1128/JB.186.7.1933-1944.2004 15028677 PMC374404

[B45] Walmagh M Boczkowska B Grymonprez B Briers Y Drulis-Kawa Z Lavigne R Characterization of five novel endolysins from Gram-negative infecting bacteriophages Appl Microbiol Biotechnol 2012 10.1007/s00253-012-4294-7 22832988

[B46] Gill JJ Summer EJ Russell WK Cologna SM Carlile TM Fuller AC Kitsopoulos K Mebane LM Parkinson BN Sullivan D Carmody LA Gonzalez CF LiPuma JJ Young R Genomes and characterization of phages Bcep22 and BcepIL02, founders of a novel phage type in *Burkholderia cenocepacia* J Bacteriol 2011 193 5300 5313 10.1128/JB.05287-11 21804006 PMC3187461

[B47] Garbe J Bunk B Rohde M Schobert M Sequencing and characterization of *Pseudomonas aeruginosa* phage JG004 BMC Microbiol 2011 11 102 10.1186/1471-2180-11-102 21569567 PMC3120641

[B48] Promega Corporation DNA Isolation From Lambda Lysates Using the Wizard® DNA Clean-Up Systemhttp://www.promega.ca/resources/articles/pubhub/enotes/dna-isolation-from-lambda-lysates-using-the-wizard-dna-cleanup-system

[B49] Drummond AJ Ashton B Buxton S Cheung M Cooper A Duran C Heled J Kearse M Markowitz S Moir R Stones-Havas S Sturrock S Swidan F Thierer T Wilson A Geneious v5.6 http://www.geneious.com

[B50] Reese MG Application of a time-delay neural network to promoter annotation in the *Drosophila melanogaster* genome Comput Chem 2001 26 51 56 10.1016/S0097-8485(01)00099-7 11765852

[B51] Lavigne R Sun WD Volckaert G PHIRE, a deterministic approach to reveal regulatory elements in bacteriophage genomes Bioinformatics 2004 20 629 635 10.1093/bioinformatics/btg456 15033869

[B52] Crooks GE Hon G Chandonia JM Brenner SE WebLogo: a sequence logo generator Genome Res 2004 14 1188 1190 10.1101/gr.849004 15173120 PMC419797

[B53] Kingsford CL Ayanbule K Salzberg SL Rapid, accurate, computational discovery of Rho-independent transcription terminators illuminates their relationship to DNA uptake Genome Biol 2007 8 R22 10.1186/gb-2007-8-2-r22 17313685 PMC1852404

[B54] Lukashin AV Borodovsky M GeneMark.hmm: New solutions for gene finding Nucleic Acids Res 1998 26 1107 1115 10.1093/nar/26.4.1107 9461475 PMC147337

[B55] Altschul SF Madden TL Schäffer AA Zhang J Zhang Z Miller W Lipman DJ Gapped BLAST and PSI-BLAST: A new generation of protein database search programs Nucleic Acids Res 1997 25 3389 3402 10.1093/nar/25.17.3389 9254694 PMC146917

[B56] Söding J Biegert A Lupas AN The HHpred interactive server for protein homology detection and structure prediction Nucleic Acids Res 2005 33 W244 W248 10.1093/nar/gki408 15980461 PMC1160169

[B57] Marchler-Bauer A Bryant SH CD-Search: Protein domain annotations on the fly Nucleic Acids Res 2004 32 W327 W331 10.1093/nar/gkh454 15215404 PMC441592

[B58] NCBI ORF Finderhttp://www.ncbi.nlm.nih.gov/projects/gorf

[B59] GenSkew – visualization of nucleotide skew in genome sequenceshttp://genskew.csb.univie.ac.at

[B60] Vincze T Posfai J Roberts RJ NEBcutter: A program to cleave DNA with restriction enzymes Nucleic Acids Res 2003 31 3688 3691 10.1093/nar/gkg526 12824395 PMC168933

[B61] Saha S Raghava GP BTXpred: prediction of bacterial toxins In Silico Biol 2007 7 405 412 18391233

[B62] Transcription Terminator Predictionhttp://pepper.molgenrug.nl/index.php/pepper-tools/terminator-predictie

[B63] Krogh A Larsson B von Heijne G Sonnhammer ELL Predicting transmembrane protein topology with a hidden Markov model: Application to complete genomes J Mol Biol 2001 305 567 580 10.1006/jmbi.2000.4315 11152613

[B64] Viklund H Elofsson A OCTOPUS: Improving topology prediction by two-track ANN-based preference scores and an extended topological grammar Bioinformatics 2008 24 1662 1668 10.1093/bioinformatics/btn221 18474507

[B65] Bendtsen JD Nielsen H von Heijne G Brunak S Improved prediction of signal peptides: SignalP 3.0 J Mol Biol 2004 340 783 795 10.1016/j.jmb.2004.05.028 15223320

[B66] Juncker AS Willenbrock H von Heijne G Brunak S Nielsen H Krogh A Prediction of lipoprotein signal peptides in Gram-negative bacteria Protein Sci 2003 12 1652 1662 10.1110/ps.0303703 12876315 PMC2323952

[B67] Silhavy TJ Berman ML Enquist LW Experiments with Gene Fusions 1984 Cold Spring Harbor Laboratory: Cold Spring Harbor

